# Unexpected formation and crystal structure of tetra­kis­(1*H*-pyrazole-κ*N* ^2^)­palladium(II) dichloride

**DOI:** 10.1107/S160053681402460X

**Published:** 2014-11-15

**Authors:** Thomas Wagner, Nena Christiansen, Cristian G. Hrib, Dieter E. Kaufmann, Frank T. Edelmann

**Affiliations:** a Chemisches Institut, Otto-von-Guericke-Universität Magdeburg, Universitätsplatz 2, D-39106 Magdeburg, Germany; b Institut für Organische Chemie, Technische Universität Clausthal, Leibnizstrasse 6, D-38678 Clausthal-Zellerfeld, Germany

**Keywords:** crystal structure, palladium(II), homoleptic metal–pyrazole complex, hydrogen bonding, solvolytic ligand degradation

## Abstract

The title salt, [Pd(C_3_H_4_N_2_)_4_]Cl_2_, was obtained unexpectedly by the reaction of palladium(II) dichloride with equimolar amounts of 1-chloro-1-nitro-2,2,2-tris­(pyrazol­yl)ethane in methanol solution. The Pd^2+^ cation is located on an inversion centre and has a square-planar coordination sphere defined by four N atoms of four neutral pyrazole ligands. The average Pd—N distance is 2.000 (2) Å. The two chloride anions are not coordinating to Pd^2+^. They are connected to the complex cations through N—H⋯Cl hydrogen bonds. In addition, C—H⋯Cl hydrogen bonds are observed, leading to a three-dimensional linkage of cations and anions.

## Abstract 2

In the title salt, the Pd^2+^ cation is located on an inversion centre and has a square-planar coordination sphere defined by four N atoms of four neutral pyrazole ligands. The two chloride anions are not coordinating to Pd^2+^ but are connected to the complex cations through N—H⋯Cl hydrogen bonds. C—H⋯Cl hydrogen bonds lead to a three-dimensional linkage of cations and anions.

## Chemical context   

**Figure 1:**
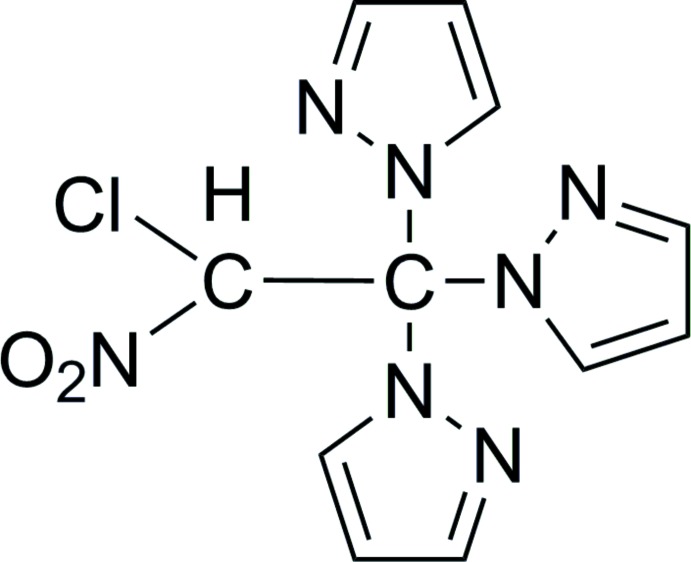
Structure diagram of the starting material 1-chloro-1-nitro-2,2,2-tris(pyrazol­yl)ethane.

Transition metal complexes containing pyrazole or substituted pyrazoles as ligands are of current inter­est due to their supra­molecular arrangements (Lumme *et al.*, 1988 ▶; Takahashi *et al.*, 2006 ▶; Casarin *et al.*, 2007 ▶; Alsalme *et al.*, 2013 ▶). In the course of an investigation on the coordination chemistry of various azolyl-nitro­chloro­alkanes (Zapol’skii & Kaufmann, 2008 ▶), we have previously studied the reaction of copper(II) perchlorate hexa­hydrate with equimolar amounts of 1-chloro-1-nitro-2,2,2-tris­(pyrazol­yl)ethane, Cl(NO_2_)CH—C(C_3_H_3_N_2_)_3_ (Fig. 1 ▶) in methanol solution (Edelmann *et al.*, 2008 ▶). Quite unexpectedly, a complete degradation of the starting material took place during the course of this reaction. As a result, the dark-blue compound *trans*-bis­(perchlorato)-tetra­kis(pyrazole)copper(II), [Cu(C_3_H_4_N_2_)_4_(ClO_4_)_2_], was isolated. The formation of free pyrazole could only be explained by a solvolytic degradation of the starting material. This degradation must have taken place to a large extent as the isolated yield was 64% (Edelmann *et al.*, 2008 ▶).

We have now carried out a closely related reaction of 1-chloro-1-nitro-2,2,2-tris­(pyrazol­yl)ethane with palladium(II) dichloride in methanol solution. Structure determination of the yellow reaction product using X-ray analysis surprisingly again revealed the presence of a homoleptic pyrazole complex. The structure of the resultant title compound, [Pd(C_3_H_4_N_2_)_4_]Cl_2_ is presented here. An elemental analysis of the title compound was also in very good agreement with the composition C_12_H_16_Cl_2_PdN_8_. In this case, too, the yield was fairly high (56%), indicating a far-reaching decomposition of the starting material. Apparently, the ligand degradation of azolyl-nitro­chloro­alkanes in the presence of transition metal salts is a more common phenomenon than originally anti­ci­pated.
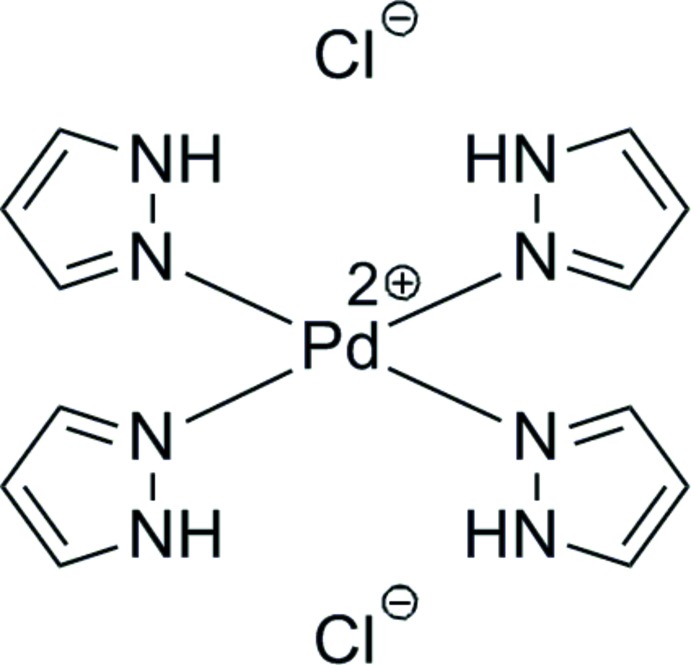


## Structural commentary   

**Figure 2:**
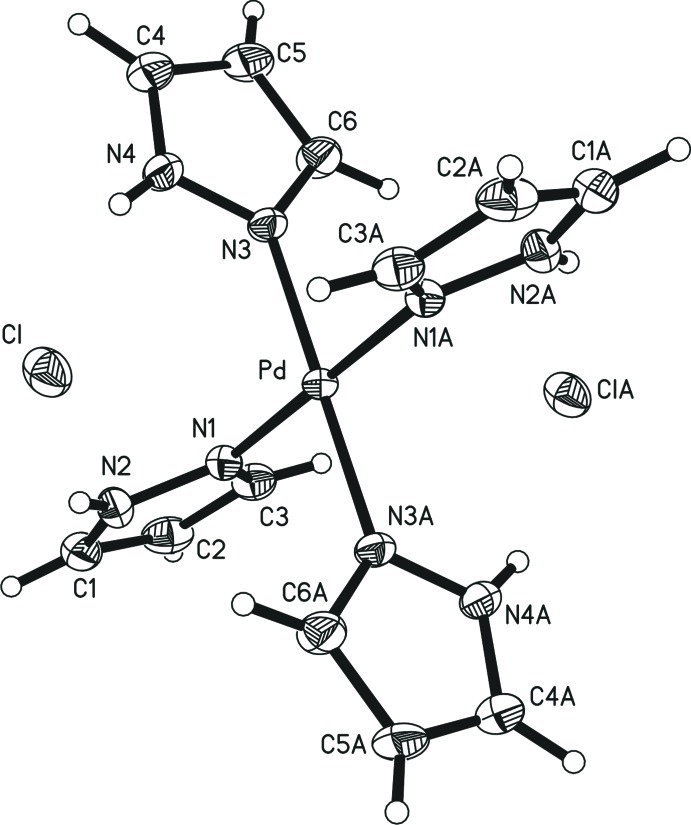
The coordination sphere of Pd^2+^ and the Cl^−^ counter-ions in the title compound. Displacement ellipsoids represent the 50% probability level. [Symmetry code (A): −*x* + 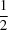, −*y* + 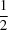, −*z*.]

In the crystal structure of the title compound, the Pd^2+^ ion is located on an inversion centre and is bonded to four neutral pyrazole ligands within a square-planar coordination environment (Fig. 2 ▶). The average Pd—N distance in the [Pd(pyrazole)_4_]^2+^ cation is 2.000 (2) Å. This is exactly the same value as found for the Cu—N distance in *trans*-bis­(perchlorato)-tetra­kis­(pyrazole)­copper(II) [2.000 (1) Å; Edelmann *et al.*, 2008 ▶]. The two chloride anions are not coordinating to the Pd^2+^ cation. This is in marked contrast to the analogous copper(II) complex [Cu(pyrazole)_4_Cl_2_] (Xing *et al.*, 2006 ▶), in which the Cu^2+^ ion is six-coordinated by four N atoms from four pyrazole ligands and two Cl^−^ ions. The same octa­hedral coordination has also been reported for the manganese(II) analog [Mn(pyrazole)_4_Cl_2_] (Lumme, 1985 ▶).

## Supra­molecular features   

**Figure 3:**
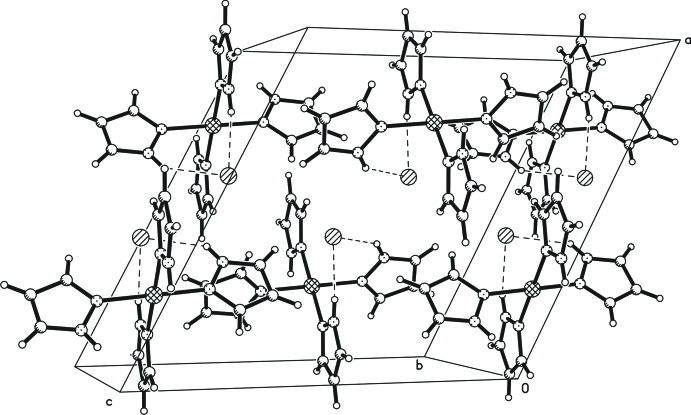
A packing diagram of the title compound. Dashed lines indicate N—H⋯Cl hydrogen-bonding inter­actions.

In the title compound, the crystal packing is stabilized by two N—H⋯Cl hydrogen bonds (Table 1 ▶) between the complex cations and the Cl^−^ counter-anions (Fig. 3 ▶). Weaker C—H⋯Cl hydrogen bonds are also observed, stabilizing a three-dimensional network. The crystal structures of the formally analogous complexes [*M*(pyrazole)_4_Cl_2_] show related features. In the structures with *M* = Mn and Cu and an octa­hedral coordination of the metal cation, the crystal structures likewise exhibit N—H⋯Cl and C—H⋯Cl hydrogen bonds which, in combination, yield three-dimensional networks.

## Relation with other compounds   

Various closely related homoleptic metal–pyrazole complexes are known from the literature (Misra *et al.*, 1998 ▶; Reedijk, 1969 ▶; Sastry *et al.*, 1986 ▶). Analogous complexes of composition [*M*(pyrazole)_4_Cl_2_] have previously been reported for *M* = Mn, Fe, Co, Ni, and Cu (Daugherty & Swisher, 1968 ▶; Bagley *et al.*, 1970 ▶; Nicholls & Warburton, 1970 ▶, 1971 ▶; Lumme, 1985 ▶; Sun *et al.*, 2001 ▶; Xing *et al.*, 2006 ▶). Generally, these compounds are prepared in a more straightforward manner by treatment of the transition metal dichlorides with four equivalents of pyrazole in suitable solvents such as methanol. While the analogous nickel(II) complex has been studied frequently (Daugherty & Swisher, 1968 ▶; Nicholls & Warburton, 1970 ▶), to the best of our knowledge neither the title compound nor the platinum homologue [Pt(pyrazole)_4_]Cl_2_ have ever been reported.

## Synthesis and crystallization   

Solid palladium(II) dichloride (0.28 g, 1.6 mmol) was added to a solution of 1-chloro-1-nitro-2,2,2-tris­(pyrazol­yl)ethane (0.50 g, 1.6 mmol) in methanol (100 ml). After stirring for 48 h at room temperature, a small amount of unreacted PdCl_2_ was removed by filtration. Crystallization from the clear filtrate at 276–279 K for 14 d afforded bright-yellow crystals of the title compound. Yield: 0.4 g (56%). Analysis calculated for C_12_H_16_Cl_2_PdN_8_: C 32.06%; H 3.59%; N 24.92%; Cl 15.77%; found: C 31.55%; H 3.38%; N 25.13%; Cl 15.25%. IR (KBr): 3090*vs*, 2977*vs*, 2371*m*, 1798*w*, 1772*w*, 1632*w*, 1518*m*, 1487*m*, 1472*s*, 1401*m*, 1367*s*, 1312*m*, 1264*m*, 1251*m*, 1209*w*, 1181*vs*, 1169*m*, 1139*s*, 1123*s*, 1078*vs*, 1052*vs*, 983*m*, 956*m*, 913*m*, 908*m*, 899*m*, 886*m*, 878*m*, 779*vs*, 739*s*, 615*s*, 606*s* cm^−1^.

## Refinement   

Crystal data, data collection and structure refinement details are summarized in Table 2 ▶. The hydrogen atoms attached to carbon were included using a riding model, with C—H = 0.95 Å, and with *U*_iso_(H) = 1.2*U*_eq_(C). The hydrogen atoms attached to nitro­gen were refined with a restrained distance N—H = 0.88 (2) Å and with *U*_iso_(H) = 1.2*U*_eq_(N).

## Supplementary Material

Crystal structure: contains datablock(s) I, New_Global_Publ_Block. DOI: 10.1107/S160053681402460X/wm5084sup1.cif

Structure factors: contains datablock(s) I. DOI: 10.1107/S160053681402460X/wm5084Isup2.hkl

CCDC reference: 1033485

Additional supporting information:  crystallographic information; 3D view; checkCIF report

## Figures and Tables

**Table 1:** Hydrogen-bond geometry (, )

*D*H*A*	*D*H	H*A*	*D* *A*	*D*H*A*
N2H2*N*Cl	0.87(2)	2.50(3)	3.254(3)	145(3)
N4H4*N*Cl	0.88(2)	2.33(2)	3.147(3)	156(4)
C1H1Cl^i^	0.95	2.75	3.625(4)	153
C4H4Cl^ii^	0.95	2.73	3.656(4)	164

Symmetry codes: (i) 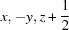; (ii) 
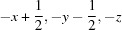
.

**Table 2:** Experimental details

Crystal data	
Chemical formula	[Pd(C_12_H_16_N_8_)]Cl_2_
*M*_r_	449.63
Crystal system, space group	Monoclinic, *C*2/*c*
Temperature (K)	150
*a*, *b*, *c* ()	13.797(3), 9.6560(19), 14.174(3)
()	117.80(3)
*V* (^3^)	1670.4(6)
*Z*	4
Radiation type	Mo *K*
(mm^1^)	1.44
Crystal size (mm)	0.40 0.40 0.20

Data collection	
Diffractometer	Stoe IPDS 2T
Absorption correction	Multi-scan (Blessing, 1995 ▶)
*T*_min_, *T*_max_	0.562, 0.750
No. of measured, independent and observed [*I* > 2(*I*)] reflections	7700, 2253, 2030
*R*_int_	0.054
(sin /)_max_ (^1^)	0.686

Refinement	
*R*[*F* ^2^ > 2(*F* ^2^)], *wR*(*F* ^2^), *S*	0.039, 0.094, 1.12
No. of reflections	2253
No. of parameters	112
No. of restraints	2
H-atom treatment	H atoms treated by a mixture of independent and constrained refinement
_max_, _min_ (e ^3^)	1.79, 1.73

Computer programs: *X-AREA* and *X-RED32* (Stoe, 2008 ▶), *SHELXS97*, *SHELXL97* and *XP* (Sheldrick, 2008 ▶).

## supplementary crystallographic information

### Crystal data

**Table:**

[Pd(C_12_H_16_N_8_)]Cl_2_	*F*(000) = 896
*M**_r_* = 449.63	449.6
Monoclinic, *C*2/*c*	*D*_x_ = 1.788 Mg m^−^^3^
Hall symbol: -C 2yc	Mo *K*α radiation, λ = 0.71073 Å
*a* = 13.797 (3) Å	Cell parameters from 15423 reflections
*b* = 9.6560 (19) Å	θ = 2.7–29.6°
*c* = 14.174 (3) Å	µ = 1.44 mm^−^^1^
β = 117.80 (3)°	*T* = 150 K
*V* = 1670.4 (6) Å^3^	Prism, yellow
*Z* = 4	0.40 × 0.40 × 0.20 mm

### Data collection

**Table:**

Stoe IPDS-2T diffractometer	2253 independent reflections
Radiation source: fine-focus sealed tube	2030 reflections with *I* > 2σ(*I*)
Graphite monochromator	*R*_int_ = 0.054
Detector resolution: 6.67 pixels mm^-1^	θ_max_ = 29.2°, θ_min_ = 2.7°
ω–scans	*h* = −18→18
Absorption correction: multi-scan (Blessing, 1995)	*k* = −13→13
*T*_min_ = 0.562, *T*_max_ = 0.750	*l* = −17→19
7700 measured reflections	

### Refinement

**Table:**

Refinement on *F*^2^	Primary atom site location: structure-invariant direct methods
Least-squares matrix: full	Secondary atom site location: difference Fourier map
*R*[*F*^2^ > 2σ(*F*^2^)] = 0.039	Hydrogen site location: inferred from neighbouring sites
*wR*(*F*^2^) = 0.094	H atoms treated by a mixture of independent and constrained refinement
*S* = 1.12	*w* = 1/[σ^2^(*F*_o_^2^) + (0.049*P*)^2^ + 4.2172*P*] where *P* = (*F*_o_^2^ + 2*F*_c_^2^)/3
2253 reflections	(Δ/σ)_max_ < 0.001
112 parameters	Δρ_max_ = 1.79 e Å^−^^3^
2 restraints	Δρ_min_ = −1.73 e Å^−^^3^

### Special details

**Table:**

Geometry. All e.s.d.'s (except the e.s.d. in the dihedral angle between two l.s. planes) are estimated using the full covariance matrix. The cell e.s.d.'s are taken into account individually in the estimation of e.s.d.'s in distances, angles and torsion angles; correlations between e.s.d.'s in cell parameters are only used when they are defined by crystal symmetry. An approximate (isotropic) treatment of cell e.s.d.'s is used for estimating e.s.d.'s involving l.s. planes.
Refinement. Refinement of *F*^2^ against ALL reflections. The weighted *R*-factor *wR* and goodness of fit *S* are based on *F*^2^, conventional *R*-factors *R* are based on *F*, with *F* set to zero for negative *F*^2^. The threshold expression of *F*^2^ > σ(*F*^2^) is used only for calculating *R*-factors(gt) *etc*. and is not relevant to the choice of reflections for refinement. *R*-factors based on *F*^2^ are statistically about twice as large as those based on *F*, and *R*- factors based on ALL data will be even larger.

### Fractional atomic coordinates and isotropic or equivalent isotropic displacement parameters (Å^2^)

**Table:**

	*x*	*y*	*z*	*U*_iso_*/*U*_eq_	
Pd	0.2500	0.2500	0.0000	0.01539 (11)	
Cl	0.09118 (6)	−0.02409 (9)	−0.06859 (6)	0.02881 (17)	
N1	0.2363 (2)	0.2373 (2)	0.1343 (2)	0.0190 (5)	
N2	0.1606 (2)	0.1607 (3)	0.1453 (2)	0.0225 (5)	
H2N	0.116 (3)	0.109 (4)	0.093 (2)	0.027*	
N3	0.36913 (18)	0.1080 (3)	0.05645 (18)	0.0189 (4)	
N4	0.3490 (2)	−0.0279 (3)	0.0486 (2)	0.0234 (5)	
H4N	0.2803 (18)	−0.054 (5)	0.015 (2)	0.028*	
C1	0.1706 (3)	0.1749 (3)	0.2435 (2)	0.0269 (6)	
H1	0.1268	0.1311	0.2702	0.032*	
C2	0.2560 (3)	0.2644 (3)	0.2984 (3)	0.0284 (7)	
H2	0.2832	0.2944	0.3702	0.034*	
C3	0.2943 (2)	0.3019 (3)	0.2264 (2)	0.0234 (5)	
H3	0.3529	0.3642	0.2412	0.028*	
C4	0.4419 (3)	−0.0999 (4)	0.0945 (3)	0.0302 (6)	
H4	0.4484	−0.1979	0.0986	0.036*	
C5	0.5262 (3)	−0.0071 (4)	0.1346 (3)	0.0316 (7)	
H5	0.6023	−0.0271	0.1721	0.038*	
C6	0.4771 (2)	0.1239 (3)	0.1089 (2)	0.0267 (6)	
H6	0.5148	0.2099	0.1263	0.032*	

### Atomic displacement parameters (Å^2^)

**Table:**

	*U*^11^	*U*^22^	*U*^33^	*U*^12^	*U*^13^	*U*^23^
Pd	0.01373 (15)	0.01505 (16)	0.01795 (15)	0.00207 (9)	0.00784 (11)	0.00096 (9)
Cl	0.0255 (3)	0.0302 (4)	0.0336 (3)	−0.0083 (3)	0.0162 (3)	−0.0104 (3)
N1	0.0183 (10)	0.0186 (12)	0.0216 (10)	0.0018 (8)	0.0105 (9)	0.0021 (8)
N2	0.0227 (11)	0.0210 (12)	0.0275 (11)	−0.0027 (9)	0.0149 (9)	−0.0005 (9)
N3	0.0160 (10)	0.0175 (11)	0.0222 (10)	0.0035 (8)	0.0080 (8)	0.0017 (8)
N4	0.0199 (10)	0.0173 (11)	0.0329 (12)	0.0025 (9)	0.0122 (9)	0.0016 (10)
C1	0.0286 (14)	0.0259 (16)	0.0322 (14)	0.0049 (12)	0.0192 (12)	0.0057 (12)
C2	0.0299 (15)	0.0337 (18)	0.0213 (13)	0.0118 (12)	0.0117 (12)	0.0023 (11)
C3	0.0202 (12)	0.0248 (14)	0.0218 (12)	0.0037 (11)	0.0069 (10)	−0.0016 (11)
C4	0.0303 (14)	0.0252 (16)	0.0391 (16)	0.0093 (12)	0.0195 (13)	0.0067 (13)
C5	0.0216 (13)	0.0325 (18)	0.0392 (16)	0.0134 (12)	0.0128 (12)	0.0094 (13)
C6	0.0158 (11)	0.0238 (15)	0.0342 (15)	0.0005 (10)	0.0063 (11)	−0.0009 (12)

### Geometric parameters (Å, º)

**Table:**

Pd—N3	1.999 (2)	N4—H4N	0.875 (19)
Pd—N3^i^	1.999 (2)	C1—C2	1.372 (5)
Pd—N1^i^	2.002 (3)	C1—H1	0.9500
Pd—N1	2.002 (3)	C2—C3	1.399 (4)
N1—C3	1.327 (4)	C2—H2	0.9500
N1—N2	1.347 (3)	C3—H3	0.9500
N2—C1	1.340 (4)	C4—C5	1.365 (5)
N2—H2N	0.869 (18)	C4—H4	0.9500
N3—C6	1.327 (4)	C5—C6	1.400 (4)
N3—N4	1.336 (3)	C5—H5	0.9500
N4—C4	1.332 (4)	C6—H6	0.9500
			
N3—Pd—N3^i^	180.00 (11)	N2—C1—C2	107.4 (3)
N3—Pd—N1^i^	89.89 (10)	N2—C1—H1	126.3
N3^i^—Pd—N1^i^	90.11 (10)	C2—C1—H1	126.3
N3—Pd—N1	90.11 (10)	C1—C2—C3	105.4 (3)
N3^i^—Pd—N1	89.89 (10)	C1—C2—H2	127.3
N1^i^—Pd—N1	180.0 (2)	C3—C2—H2	127.3
C3—N1—N2	106.7 (2)	N1—C3—C2	109.7 (3)
C3—N1—Pd	128.7 (2)	N1—C3—H3	125.2
N2—N1—Pd	124.51 (19)	C2—C3—H3	125.2
C1—N2—N1	110.8 (3)	N4—C4—C5	107.4 (3)
C1—N2—H2N	130 (3)	N4—C4—H4	126.3
N1—N2—H2N	119 (3)	C5—C4—H4	126.3
C6—N3—N4	107.2 (2)	C4—C5—C6	105.7 (3)
C6—N3—Pd	130.1 (2)	C4—C5—H5	127.2
N4—N3—Pd	122.71 (18)	C6—C5—H5	127.2
C4—N4—N3	110.9 (3)	N3—C6—C5	108.8 (3)
C4—N4—H4N	132 (3)	N3—C6—H6	125.6
N3—N4—H4N	117 (3)	C5—C6—H6	125.6
			
N3—Pd—N1—C3	84.2 (3)	N1—Pd—N3—N4	83.8 (2)
N3^i^—Pd—N1—C3	−95.8 (3)	C6—N3—N4—C4	−0.4 (3)
N1^i^—Pd—N1—C3	−138 (100)	Pd—N3—N4—C4	−178.5 (2)
N3—Pd—N1—N2	−97.9 (2)	N1—N2—C1—C2	0.1 (3)
N3^i^—Pd—N1—N2	82.1 (2)	N2—C1—C2—C3	0.4 (3)
N1^i^—Pd—N1—N2	40 (100)	N2—N1—C3—C2	0.9 (3)
C3—N1—N2—C1	−0.6 (3)	Pd—N1—C3—C2	179.0 (2)
Pd—N1—N2—C1	−178.9 (2)	C1—C2—C3—N1	−0.8 (3)
N3^i^—Pd—N3—C6	127 (100)	N3—N4—C4—C5	0.4 (4)
N1^i^—Pd—N3—C6	86.1 (3)	N4—C4—C5—C6	−0.3 (4)
N1—Pd—N3—C6	−93.9 (3)	N4—N3—C6—C5	0.2 (4)
N3^i^—Pd—N3—N4	−55 (100)	Pd—N3—C6—C5	178.1 (2)
N1^i^—Pd—N3—N4	−96.2 (2)	C4—C5—C6—N3	0.1 (4)

Symmetry code: (i) −*x*+1/2, −*y*+1/2, −*z*.

### Hydrogen-bond geometry (Å, º)

**Table:**

*D*—H···*A*	*D*—H	H···*A*	*D*···*A*	*D*—H···*A*
N2—H2*N*···Cl	0.87 (2)	2.50 (3)	3.254 (3)	145 (3)
N4—H4*N*···Cl	0.88 (2)	2.33 (2)	3.147 (3)	156 (4)
C1—H1···Cl^ii^	0.95	2.75	3.625 (4)	153
C4—H4···Cl^iii^	0.95	2.73	3.656 (4)	164

Symmetry codes: (ii) *x*, −*y*, *z*+1/2; (iii) −*x*+1/2, −*y*−1/2, −*z*.
